# Nanoencapsulation as a novel delivery approach for therapeutic applications of gla-rich protein (GRP)

**DOI:** 10.1080/07853890.2021.1896900

**Published:** 2021-09-28

**Authors:** Nuna Araújo, Carla Viegas, Jorge F. Pontes, Catarina Marreiros, Pedro Raimundo, Anjos L. Macedo, António Alves de Matos, Ana Grenha, Cees Vermeer, Dina Costa Simes

**Affiliations:** a Centre of Marine Sciences, University of Algarve, Algarve, Portugal; b GenoGla Diagnostics, CCMAR, University of Algarve, Algarve, Portugal; c Centre for Biomedical Research, University of Algarve, Algarve, Portugal; d Departamento de Ciências Biomédicas e Medicina, Universidade do Algarve, Algarve, Portugal; e UCIBIO-REQUIMTE, Departamento de Química, Faculdade de Ciências e Tecnologia, Universidade NOVA de Lisboa, Caparica, Portugal; f Centro de Investigação Interdisciplinar Egas Moniz (CiiEM), Egas Moniz Cooperativa de Ensino Superior, Caparica, Almada, Portugal; g Maastricht University, Maastricht, The Netherlands

## Abstract

**Introduction:**

Gla rich protein (GRP) is a vitamin K dependent protein, shown to function as an inhibitor of pathological calcification and as an anti-inflammatory agent, with potential therapeutic use for age-related diseases such as osteoarthritis (OA) [1,2]. OA is a leading cause of disability and morbidity in the older population and constitutes a major worldwide challenge for our health system. Presently, there are no drugs approved that can prevent, stop, or even restrain progression of OA. GRP has been shown to be able to lower inflammation and mineralisation processes in the articular tissue. Chitosan/tripolyphosphate (TPP) nanoparticles were selected for this study due to their biocompatibility, biodegradability and capacity to overcome the problem of low solubility of GRP in physiological conditions. This study aims to produce and characterise chitosan/TPP nanoparticles as GRP-delivery vehicles and test its anti-inflammatory potential in human macrophages.

**Materials and methods:**

Nanoparticles of fluorescein-labelled chitosan/TPP with and without GRP (NG and NP, respectively) were prepared by ionic gelation [3]. Resulting NP and NG were characterised by dynamic light scattering, transmission electron microscopy (TEM) and flow cytometry. The anti-inflammatory activity of NP and NG was assessed in THP-1 cells differentiated to macrophages. Mac-THP-1 cells were pre-treated with both NP and NG, followed by LPS stimulation. Cell viability was assessed by the MTS cell proliferation assay, and levels of TNFα released to cell culture media were determined by ELISA.

**Results:**

The average size determined for NG was increased relatively to the NP, while flow cytometry and TEM analysis indicate the presence of GRP in NG, suggesting an effective incorporation of human recombinant GRP. Flow cytometry studies confirmed the cellular uptake of nanoparticles by macrophages. The GRP-loaded nanoparticles were able to reduce the production of TNFα in LPS-stimulated macrophages.

**Discussion and conclusions:**

The results confirm that chitosan/TPP nanoparticles are excellent drug delivery vehicles for GRP in macrophages and predict a wider therapeutic application in chronic inflammation-related diseases. GRP-containing nanoparticles will be further used in OA functional assays and the results will bring new knowledge on the role of GRP in the interplay between inflammation and mineralisation events associated with OA.
